# Function of HP1 proteins as a component in kinetochore formation and its relation with chromosome instability

**DOI:** 10.1186/1756-8935-6-S1-P22

**Published:** 2013-03-18

**Authors:** Rodrigo González-Barrios, Ernesto Soto-Reyes, Julia Medoza Perez, Alejandro Lopez-Saavedra, Clementina H Castro, Luis A Herrera

**Affiliations:** 1 Unidad de Investigación Biomédica en Cáncer, Instituto Nacional de Cancerología (INCan)-Instituto de Investigaciones Biomédicas (IIB), Universidad Nacional Autónoma de México (UNAM), Mexico City, Mexico

## 

HP1 Family of proteins are involved in the formation and maintenance of chromatin higher order structure. In mammals there are known three isotypes (HP1α, HP1β and HP1γ). Recently, it has been proposed that HP1 may play an important roll in inner centromere establishment, generated by its interaction with HMis12 complex, (HMis12C) which is relevant in kinetochore formation and microtubule recognition which ensure correct chromosomal segregation. However, alterations in chromatin structure or loss in H3K9 methylation lead to a reduction of the protein presence and changes of HP1 proteins localization to heterochromatin followed by chromosome instability. It has not been studied if this is mediated by loss of recruitment of HMis12C to the kinetochore and which is it relation with chromosomal instability generation. Thus, the aim of this study is to determine if alteration of HP1 proteins is capable of reducing HMis12C recruitment to the kinetochore. We elaborated transfected of constructions of HP1-GFP for each isotype in HCT116 cells and performed time-lapse to observe localization along cell cycle by confocal microscopy; in addition, we treated cells with TSA 1uM to analyze changes in HP1 localization. We used ChIP assay in satellite alpha and satellite 2 to determine presence of H3H9me3, HP1 proteins, CENPA, and HMis12 in normal HCT116 and in HCT116 transfected cells with HP1-GFP and with Jmjd2b to observe the effect of the loss of H3K9me3 to HMis12C incorporation. We found that each isotype present a different localization at interphase, but HP1α and β are present at the centromere at this fase, also this localization is highly dynamic in mitosis where HP1β is removed and HP1α is enriched at the chromosomes centromere. Treatment with TSA increases chromosome instability and generates relocalization of HP1 proteins to pericentromeric chromatin where H3K9me3 remains and propagates. Jmjd2b over-expression reduces HP1 presence at chromatin and also reduces HMis12 in mitosis. These results support another function of HP1 as a kinetochore partner leading incorporation of HMis12 during cell division.

This work was supported by CONACYT 83959 and PAPIITIN213311

